# Manifestation of the interplay between spin–orbit and Jahn–Teller effects in Au_25_ superatom UV-Vis fingerprint spectra†

**DOI:** 10.1039/d3sc00944k

**Published:** 2023-04-11

**Authors:** Can Liao, Manzhou Zhu, De-en Jiang, Xiaosong Li

**Affiliations:** a Department of Chemistry, University of Washington Seattle WA 98195 USA xsli@uw.edu; b Department of Chemistry and Center for Atomic Engineering of Advanced Materials, Anhui University China zmz@ahu.edu.cn; c Department of Chemical and Biomolecular Engineering, Vanderbilt University Nashville TN 37235 USA de-en.jiang@vanderbilt.edu

## Abstract

Atomically precise nanoclusters play an important role in nanoscale catalysis, photonics, and quantum information science. Their nanochemical properties arise from their unique superatomic electronic structures. As the flagship of atomically precise nanochemistry, the Au_25_(SR)_18_ nanocluster exhibits tunable spectroscopic signatures that are sensitive to the oxidation state. This work aims to unravel the physical underpinnings of the spectral progression of Au_25_(SR)_18_ nanocluster using variational relativistic time-dependent density functional theory. The investigation will focus on the effects of superatomic spin–orbit coupling, its interplay with Jahn–Teller distortion, and their manifestations in the absorption spectra of Au_25_(SR)_18_ nanoclusters of different oxidation states.

## Abstract 2

Atomically precise nanoclusters play an important role in nanoscaled catalysis, photonics, and quantum information science.

## Graphical abstract

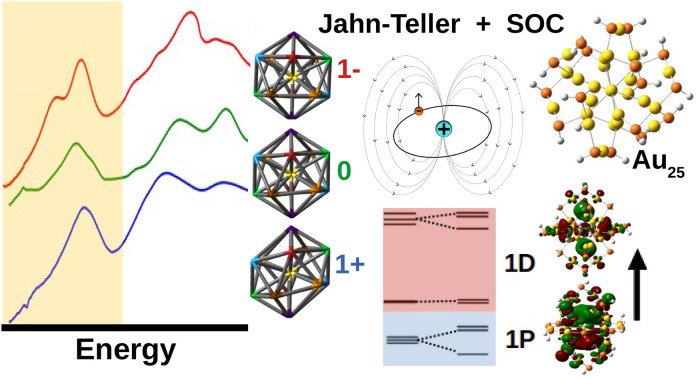

The Au_25_(SR)_18_ nanocluster is the flagship of atomically precise nanochemistry.^1^ Over one thousand papers have been published about it and its derivatives since its identification in 2005 (ref. 2) and structure determination in 2008.^1,3–7^ However, the influence of spin–orbit coupling (SOC) over different oxidation states of the Au_25_(SR)_18_ nanocluster has not been fully understood, despite it being the most studied cluster in the field and recent attempts.^8–11^ SOC plays an important role in the electronic structure and properties of molecules and materials, especially for heavier elements such as Au. The inclusion of SOC in *ab initio* calculations of ground and excited state nanomaterials has not been routinely pursued due to the high computational cost. This challenge has prevented a full understanding of some of the most studied nanosystems.

Fig. 1 shows the experimental UV-Vis absorption spectra of Au_25_(SR)_18_ nanoclusters (abbreviated as Au_25_) at three different oxidation states. The differences among them can clearly be seen. The low energy region from 1.2 eV to 2.0 eV, known as the fingerprint band, corresponds mainly to the transitions between frontier orbitals: the double peak in Au_25_^−^ changes to an asymmetric band in neutral Au_25_ with further blue-shifts in Au_25_^+^.^12–15^ The frontier orbitals of Au_25_ can be well-understood in the absence of SOC by the superatomic complex model:^16^ Au_25_^−^ being an 8-electron system with a superatomic electron configuration of (1S)^2^(1P)^6^, with 1D orbitals being the LUMO. Although the double peaks of Au_25_^−^ were explained by the SOC splitting of the 1P orbitals based on two-component time-dependent density functional theory (2C-TDDFT),^8^ the role of SOC in the neutral and cationic spectra remains unexplored.

**Fig. 1:**
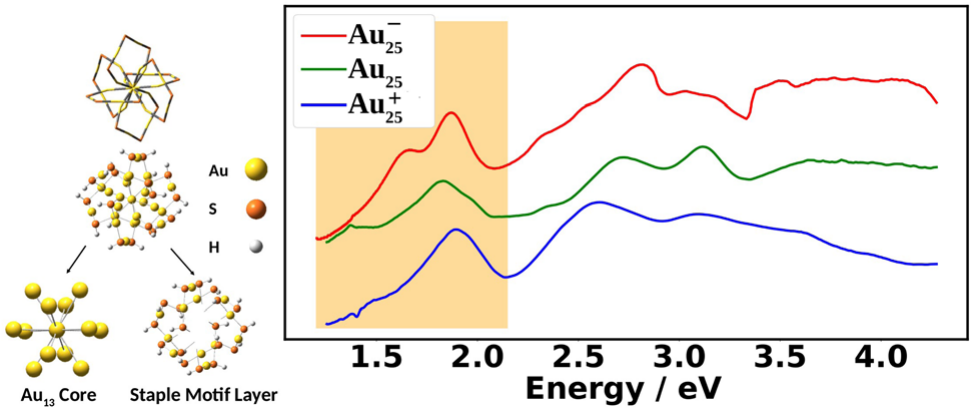
On the left, ball-and-stick and wire frame models of Au_25_(SR)_18_ are shown. The nanocluster can be separated into two regions: the Au_13_ core and the staple motif layer. In our computational model, we use R = H to reduce computational cost. On the right, the experimental UV-Vis absorption spectra of Au_25_(SPET)_18_ (SPET = 2-phenylethanethiolate: SCH_2_CH_2_Ph) at the −1, 0, and +1 charge states recorded at 78 K is shown. The shaded region is the “fingerprint” band, which is the focus of this work.

Another consideration is the interplay between geometry and electronic structure as a result of SOC. Jahn–Teller distortion plays a crucial role in the electronic structure of metal nanoclusters including ligand-protected metal clusters.^15,17,18^ It is well-known that filling degenerate or near-degenerate orbitals with unpaired electrons is met by geometric distortions that lower the nanocluster symmetry and break orbital degeneracies. Metal nanoclusters tend to not follow Hund's rule since the energy lowered by Jahn–Teller distortion can be greater than that by Hund's spin–spin coupling. This perspective is paramount to understanding the evolution of the UV-Vis fingerprint band with respect to charge. As the nanocluster oxidizes from anionic (−1) to neutral (0) and cationic (+1) oxidation states, the superatomic orbital electron configuration changes from 1P^6^ to 1P^5^ and 1P^4^, respectively, presenting a clear opportunity for Jahn–Teller distortion to act. Indeed, the Jahn–Teller effects have been shown to be present in Au_25_ which can be further manipulated to tune its redox properties.^15,19^

The goal of the present work is, therefore, to fully understand the role of SOC in the fingerprint absorption band at 1.2 eV to 2.0 eV for the −1, 0, and +1 oxidation states of the Au_25_(SR)_18_ nanocluster and to correlate the SOC-induced electron structure modulation with Jahn–Teller distortion. Au_25_(SH)_18_ was used as a model in the study to reduce computational cost since it has been shown that optical properties in this energy region are insensitive to ligand choice.^12,20,21^ All calculations were performed using a developmental version of the Gaussian electronic structure package.^22^ Two-component time-dependent density functional theory calculations were performed using the PBE0,^23,24^ functional with the relativistic CRENBL^25–29^ effective core potential (ECP) including SOC and its complementary basis set for Au and S atoms. See ESI† for more details.

Fig. 2 compares the simulated spectra with the experiment for the three different oxidation states of Au_25_. The low energy satellite peak at 1.64 eV was labeled as α_1_ and the main peak at 1.91 eV was labeled as α_2_ in Fig. 2a for the anionic cluster. Upon oxidation of the anion to the neutral nanocluster, the satellite peak lowered in intensity and was red-shifted to 1.43 eV, labeled as β_1_ in Fig. 2b. Broadening of the main peak was observed along with a slight red-shift to 1.82 eV, labeled as β_3_ in Fig. 2b. β_3_ exhibited a low energy tail with significant intensity at 1.58 eV that connects β_1_ and β_3_, labeled as β_2_ in Fig. 2b. β_3_ also exhibited a high energy shoulder, labeled β_4_. Further oxidation to the cationic state resulted in the disappearance of the low energy satellite peak along with a blue-shift of the main peak to 1.89 eV, labeled γ_2_. The low-energy tail remains at 1.70 eV and is labeled γ_1_. The high energy shoulder of the main peak is no longer present in the experimental spectrum, but remains in the simulated spectrum. Overall, one can see an excellent agreement between the simulations and the experimental spectra for the three different charge states.

**Fig. 2:**
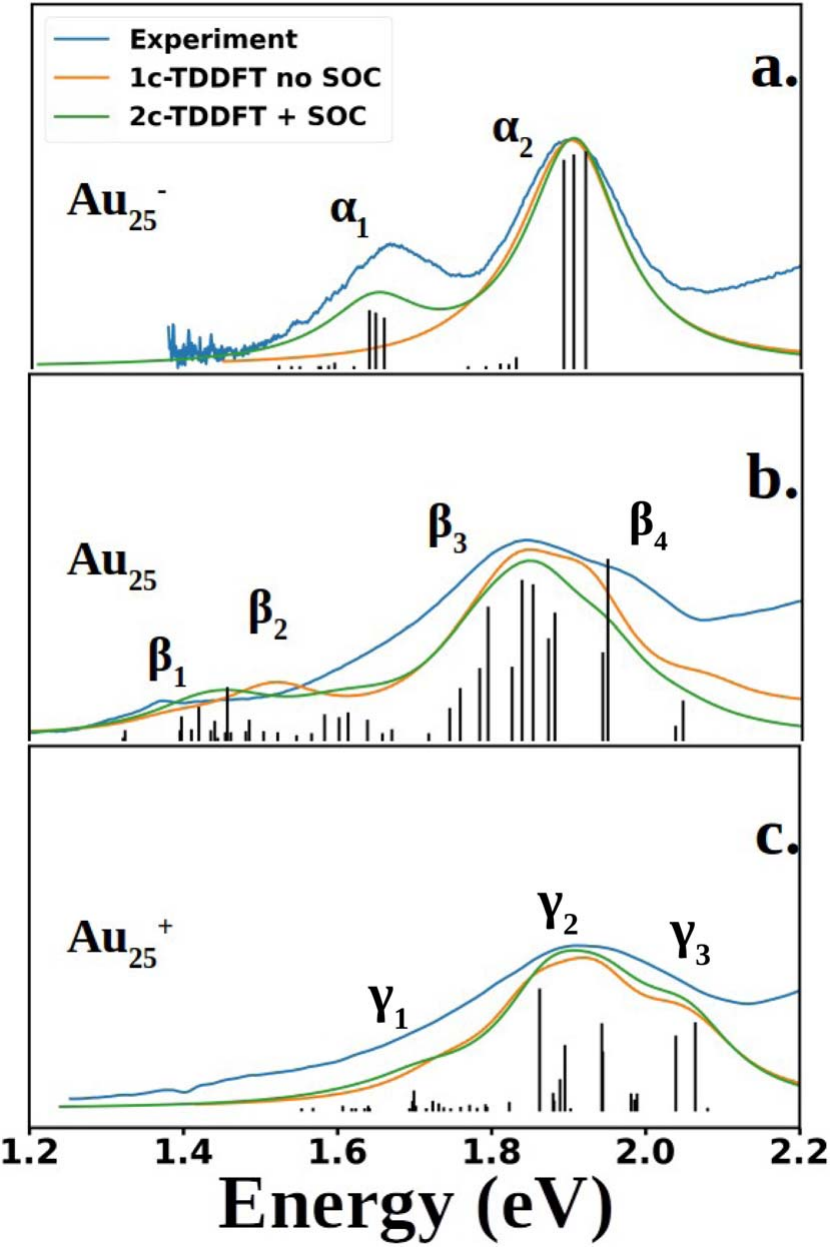
Experimental and computed spectra of Au_25_(SR)_18_. The excited states obtained from 2C-TDDFT are plotted as black lines. Spectra were generated by applying Lorentzian broadening to the excited states with a half-width at half max of 0.07 eV. The anion spectrum (a) is compared to the experimental spectrum of Au_25_(C_6_H_13_)_18_^−^ (SCH_6_H_13_: 1-hexanethiolate), reproduced from data obtained from Ramakrishna *et al.*^30^ The neutral (b) and cation (c) spectra are compared to experimental spectra of Au_25_(SPET)_18_ and Au_25_(SPET)_18_^+^ respectively. All spectra were recorded at 78 K. The computed neutral spectrum was red-shifted by 0.11 eV to align with the experimental spectrum.

We next correlate the change in the optical absorption spectra to the change in the geometry of the Au_25_ nanocluster as the charge is varied, with a focus on SOC electronic structure. We note that Jahn–Teller distortion in Au_25_ nanoclusters has been correlated to experimental optical absorption and voltammograms.^15,18,31^Fig. 3 shows the change in the nanocluster Au_13_ core geometry and diagonal lengths for each oxidation state. The anion core was nearly a perfect icosahedron. The slight imperfections left the core with a *T*_h_ symmetry rather than the *I*_h_ symmetry expected of a perfect icosahedron. Oxidation of the anion to the neutral nanocluster resulted in a distortion of the core lowering its symmetry to *C*_i_. The distortion appeared as a tilting of the 10 equatorial triangular faces of the icosahedron caused by the increase in bond angles between the purple, blue, and orange diagonals. In addition, every diagonal decreased in length leading to an overall shrinking of the core. Much greater distortion was observed when oxidized to the cation. The symmetry of the cation core remained *C*_i_ but the symmetry deviated further from icosahedron. The equatorial tilting of the icosahedron became much more pronounced as the bond angles between the purple, blue, and orange diagonals further increased. The length of each diagonal also increased with most diagonals surpassing their anionic lengths.

**Fig. 3:**
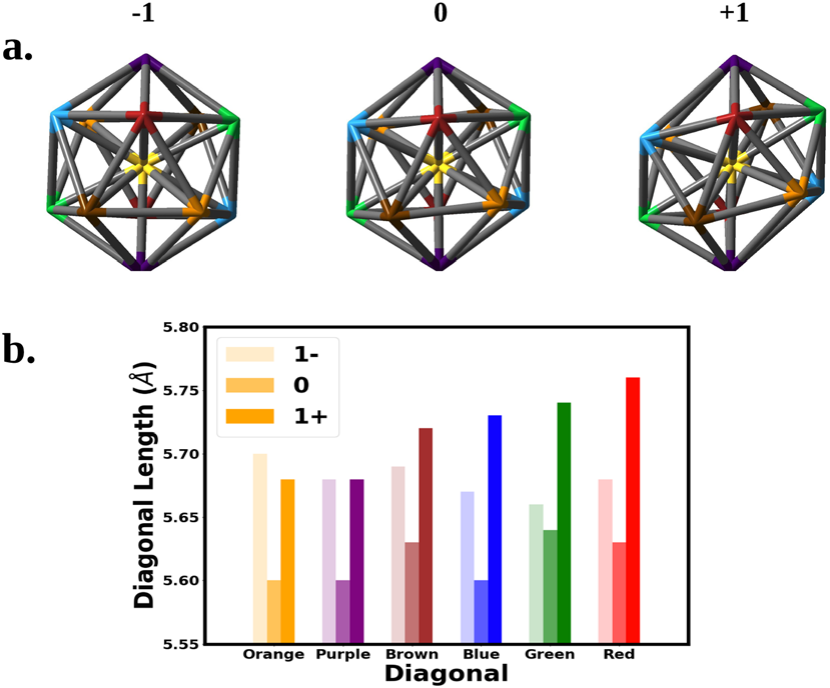
The structure of the Au_13_ core at the −1, 0, and +1 oxidation states are shown above. The vertices represent Au atoms. Atoms of the same color share a diagonal that runs through the center. The diagonal lengths are plotted for each oxidation state in the bottom chart.

Molecular orbital analysis is necessary in the characterization of the absorbance spectra. Fig. 4 shows the anionic MO diagram calculated with and without SOC along with visualization of the superatomic orbitals. The highest occupied and unoccupied orbitals are the 1P and 1D, respectively. The 1P orbitals generally have the same appearance but lie on different axes. The 1D superatomic orbitals were more diverse in their appearance including having a *d*_*z*^2^_ or “four-leaf clover” center. Because superatomic orbitals can be identified with molecular orbitals, molecular symmetry dictates how atomic spin–orbit coupling collectively manifests in superatomic orbitals.

**Fig. 4:**
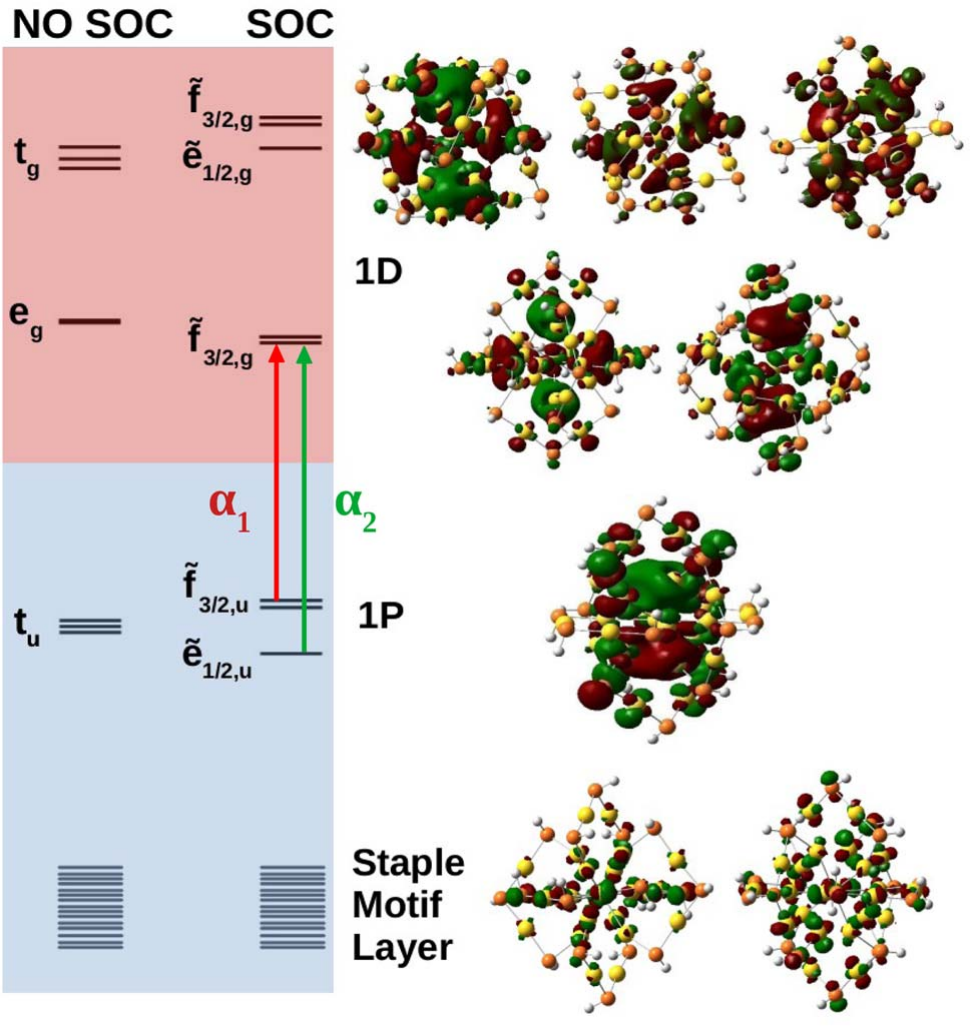
MO diagram of Au_25_(SH)_18_^−^ with and without SOC, where each line is a Kramers pair. Blue and red shaded orbitals denote occupied and unoccupied orbitals, respectively. Below the superatomic orbitals exists a high density of states region where electron density is delocalized onto the staple motif layer. Images of the orbitals are shown on the right.

The anion superatomic orbitals split in the same manner as atomic orbitals in a *T*_h_ field as shown in Fig. 4. Double group symmetry is used in the discussion of molecular orbitals under SOC. Irreducible representations (irrep) of the double symmetry is denoted with a tilde. In the discussion of orbital degeneracies, we take the perspective of spin-orbitals (*e.g.*, a non-relativistic hydrogen 2p manifold is a six-fold degeneracy). Without SOC, the 1D superatomic orbitals split into t_g_ and e_g_ while the 1P superatomic orbitals remain degenerate transforming as t_u_. With SOC, the t_g_ manifold splits into a doubly degenerate ẽ_1/2,g_ and a four-fold degeneracy f̃_3/2,g_ while the e_g_ manifold remains as a four-fold degeneracy (re-designated as f̃_3/2,g_ under double group theory). The t_u_ manifold splits into a four-fold degeneracy f̃_3/2,u_ and a doubly degenerate ẽ_1/2,u_. The relativistic field splitting was consistent with the predictions of double group theory. Below the 1P superatomic orbitals was a dense region with orbitals delocalized onto the staple motif layer. It was observed that these orbitals do play a key role in the neutral and cationic spectra within the fingerprint region.

The MO diagram of the neutral and cationic nanoclusters with SOC are shown in Fig. 5 (see ESI† for MO diagrams without SOC). The driver of Jahn–Teller distortion was the removal of electrons from the former 1P f̃_3/2,u_ manifold that breaks the spherical symmetry of the 1P shell. The nanocluster symmetry for both oxidation states lowered to *C*_i_ to break the partially filled f̃_3/2,u_ degeneracy. The neutral cluster experienced minor distortion since the destabilized 1P was still singly occupied. This was reflected in the reminiscence of the large energy gap between the former 1D f̃_3/2,g_ and ẽ_1/2,g_ manifolds, resembling *T*_h_ orbital splitting. The distortion was much greater for the cation since the depopulated 1P can further destabilize without raising overall energy. The 1D superatomic orbitals further separate closing the large energy gap. In both oxidation states, the 1P degeneracy was broken.

**Fig. 5:**
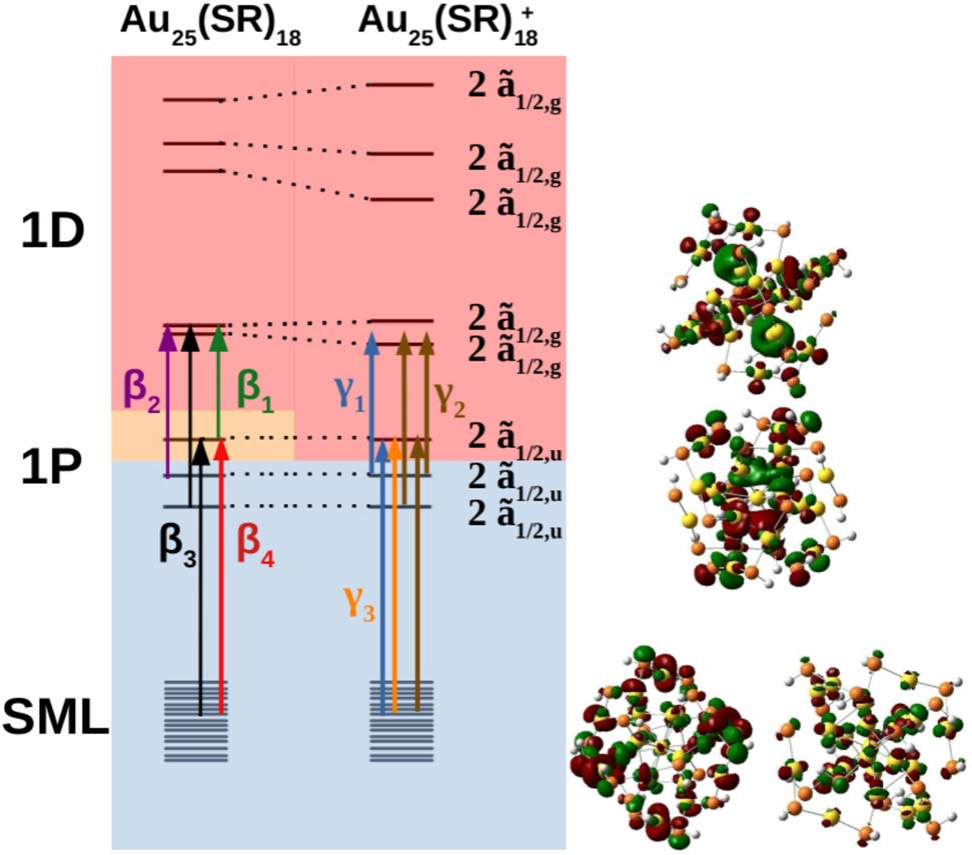
MO diagram depicting the Jahn–Teller distortion from the neutral Au_25_(SR)_18_ to Au_25_(SR)_18_^+^ where each line is a Kramers pair. Orbital transitions responsible for features β_1_, β_2_, β_3_, and β_4_ on the neutral spectrum; and γ_1_, γ_2_, and γ_3_ on the cationic spectrum, are shown. Blue, orange, and red shaded orbitals are doubly-occupied, singly-occupied, and unoccupied respectively. The double group irrep of the orbitals are shown on the right and are the same for both oxidation states. The “2” in front of the irrep indicates that the Kramers pair is a pair of spin–orbitals that transform as the given irrep (*e.g.*, 2ã_1/2,u_ indicates that two spin–orbitals that transform as ã_1/2,u_ make up the Kramers pair).

Shown in Fig. 2 are the experimental and simulated spectra for the three oxidation states of Au_25_. The most straightforward spectrum is the anionic spectrum shown in Fig. 2a. The anion spectrum consisted of two peaks, α_1_ and α_2_. The orbital excitations responsible for α_1_ and α_2_ were 1P ẽ_1/2,u_ → 1D f̃_3/2,g_ and 1P f̃_3/2,u_ → 1D f̃_3/2,g_, respectively. From the density of state point of view, α_2_, arising from four-fold degenerate f̃_3/2,u_ orbitals, should have a higher intensity than α_1_, which arises from two-fold degenerate ẽ_1/2,u_ orbitals. However, this is not the case in experiment nor our calculations. Spin projection of 2C-TDDFT results shows that the excited states responsible for α_2_ are comprised mostly of singlet character whereas those responsible for α_1_ are comprised mostly of triplet character, as shown in Table 1. Given that the ground state is a closed-shell singlet, the discrepancy in peak intensities is clearly due to the spin selection rule (Δ*S* = 0).

**Table 1:** Percentage of triplet and singlet eigenstate contribution to excited states responsible for α_1_ and α_2_ in the anion spectrum. Excited states are labeled by increasing energy within a given peak

Peak	Excited state	% triplet	% singlet
α_1_	1	74.91%	25.09%
	2	67.22%	32.78%
	3	76.65%	23.35%
α_2_	1	34.03%	65.97%
	2	26.37%	73.63%
	3	37.74%	72.26%

The neutral nanocluster spectrum is shown in Fig. 2b. Many more excited states were responsible for the neutral spectrum than for the anionic spectrum. The features β_1_ and β_2_ were direct consequences of Jahn–Teller distortion. Recall that Jahn–Teller distortion breaks the f̃_3/2,u_ degeneracy into a stabilized doubly-occupied 1P and a destabilized singly-occupied 1P. Orbital excitations from the destabilized singly-occupied 1P to the lowest two 1D gave rise to β_1_. Orbital excitations from the stabilized doubly-occupied 1P to the lowest two 1D gave rise to β_2_. Along with excitations between superatomic orbitals, the vacancy in 1P opened opportunities for the low energy orbitals delocalized onto the staple motif layer (SML) to excite into the core-localized 1P, suggesting possible ligand–metal charge transfer (LMCT) in the fingerprint region. Mixing of these orbital excitations were pervasive in the excited states throughout the spectrum. Along with orbital excitations from the lowest 1P to the lowest two 1D, SML → 1P LMCT excitations also contributed to electronic transitions that form β_3_. The rise of SML → 1P LMCT excitations with considerable oscillator strength also led to the broadening of β_3_ and the rise of β_4_. Because ligands in the theoretical model differ from experiment (R = SH as opposed to SPET), it is reasonable for the theoretical spectrum to disagree with experiment at β_4_.

The simulated cation spectrum is shown in Fig. 2c. With the depletion of the highest 1P, β_1_ was not present in the cationic spectrum, resulting in a single broad peak, labeled γ_2_, and a low energy tail, labeled γ_1_. Excitations between the middle 1P and the two lowest 1D along with SML → 1P LMCT excitations were observed in γ_1_. The high intensity excited state at 1.82 eV that constitutes γ_2_ also consists mainly of excitations between the middle 1P and the two lowest 1D. The rest of the excited states that constitute γ_2_ were from orbital excitations between the lowest 1P and the two lowest 1D along with SML → 1P LMCT. The blue-shift of the main peak compared to the neutral spectrum arose from further destabilization of the depleted 1P. Such destabilization increased the energy of excited states involving SML → 1P LMCT, leading to a blue-shift of the main peak. The lack of high intensity excited states at γ_3_ due to a minimal ligand caused the theoretical spectrum to exhibit a high energy shoulder instead of a single broad peak encompassing both γ_2_ and γ_3_, as seen in experiment.

## Conclusion

Herein, relativistic 2C-TDDFT with a variational treatment of SOC was employed to investigate the physical underpinnings behind the spectral progression of the Au_25_ nanocluster UV-Vis absorption spectrum as it oxidizes from −1 to 0 and +1 oxidation states. In addition to reproducing absorption spectra with high accuracy, the effect of the interplay between spin–orbit and Jahn–Teller distortion was carefully analyzed. To our knowledge, this was the first study to provide a thorough picture of the Au_25_ nanocluster relating SOC, Jahn–Teller distortion, and spectroscopic characteristics.

## Data availability

Data are available from the corresponding authors upon request.

## Author contributions

X. Li, D. Jiang, and M. Zhu conceived the project; C. Liao performed the computational study and data analysis; M. Zhu conducted experiments; X. Li was responsible for acquisition of funding; all authors contributed to the development of the manuscript.

## Conflicts of interest

There are no conflicts to declare.

## Supplementary Material

SC-014-D3SC00944K-s001
