# Supplementary material for: Manifestation of the interplay between spin–orbit and Jahn–Teller effects in Au25 superatom UV-Vis fingerprint spectra
Source: Chem Sci. 2023 Apr 11;14(18):4666–71. doi: 10.1039/d3sc00944k (PMC10171181; doi:10.1039/d3sc00944k)
Supplement: SC-014-D3SC00944K-s001 [file SC-014-D3SC00944K-s001.pdf]

Electronic Supplementary Material (ESI) for Chemical Science.
This journal is © The Royal Society of Chemistry 2023

Supporting Information for "Manifestation of the Interplay between Spin-Orbit and Jahn-Teller Effects in Au_{25} Superatom UV-Vis Fingerprint Spectra"

Can Liao,[†] Manzhou Zhu,[*,‡] De-en Jiang,[*,¶] and Xiaosong Li[*,†]

†*Department of Chemistry, University of Washington, Seattle, WA, 98195*

‡*Department of Chemistry and Center for Atomic Engineering of Advanced Materials, Anhui University, China*

¶*Department of Chemical and Biomolecular Engineering, Vanderbilt University, Nashville, TN, 37235*

E-mail: zmz@ahu.edu.cn; de-en.jiang@vanderbilt.edu; xsli@uw.edu

S1 Experimental Details

The $Au_{25}SPET_{18}$ nanoclusters at the −1, 0, and +1 oxidation states were obtained using methods found in the literature.[1–3]

S1.1 Preparation of the $Au_{25}SPET_{18}^-$ nanocluster

0.4 mmol of $HAuCl_4 \cdot 3H_2O$ (0.1576 g) dissolved in 5 mL water and 0.47 mmol of TOAB (0.2558 g) dissolved in 10 mL toluene were combined in a 25 mL tri-neck round bottom flask. The solution was vigorously stirred (1100 rpm) with a magnetic stir bar to facilitate

the phase transfer of the Au(III) ions into the toluene phase. After 15 minutes, phase transfer was completed, leaving a clear aqueous phase at the bottom of the flask. The aqueous solution was removed with a 10 mL syringe. The toluene solution of Au(III) was purged with N_2 and cooled to 0 °C in an ice bath for 30 minutes under magnetic stirring. 1.2 mmol of $PhCH_2CH_2SH$ (0.17 mL) was added to the reaction, and stirring was reduced to 30 rpm. The deep red solution turned faint yellow after 5 minutes and finally to clear over 1 hour. AFter the solution turned to clear, the stirring speed was changed to 1100 rpm, and immediately, an aqueous solution of $NaBH_4$ (0.1550 g, 4 mmol, freshly made in 7 mL ice-cold water) was quickly added all at once. The reaction was allowed to proceed under N_2 atmosphere. After 12 hours, the aqueous layer at the bottom of the flask was removed using a syringe. The toluene solution was dried. The toluene solution was washed with 20 mL of CH_3OH three times. After the wash, 20 mL of CH_3CN was added to separate the obtained $Au_{25}SPET_{18}^{-1}$.

S1.2 Preparation of the $Au_{25}SR_{18}$ nanocluster

5 mg of the as-prepared $Au_{25}SPET_{18}^{-}$ nanocluster was first dissolved in 20 mL of toluene and further reacted with 2 mL of H_2O_2 solution (33%, aqueous). After 20 minutes, the product was separated by precipitation with ethanol and recrystallized in a mixture of toluene and ethanol.

S1.3 Preparation of the $Au_{25}SR_{18}^{+}$ nanocluster

3 mg of the as-prepared $Au_{25}SR_{18}^{-}$ was first dissolved in 20 mL of toluene. 2 molar ratios of $TEMPO^{+}$ ($TEMPO^{+}BF_4^{-}$ dissolved in 5 mL of acetonitrile) was added using a syringe under ambient conditions. After 20 minutes, the product was separated by precipitation with ethanol and recrystallized in a mixture of toluene and ethanol.

S1.4 UV-Vis Recording

All experimental spectra were recorded at 78 K by dissolving the corresponding nanocluster in 2-methyltetrahydrofuran and the concentration was set at 1 mg/mL (0.135 M). All UV-Vis absorption spectra were recorded using an Agilent 8453 diode array spectrometer by placing 3 mL of the nanocluster solution into a 1 cm path quartz cell. The 78 K testing environment was created using a low-temperature accessory of the spectrometer assisted by liquid nitrogen.

S2 Computational Details

All calculations were performed using the same effective core potentials (ECPs), DFT functionals, and basis sets in a two-component spinor framework. All Au and S atoms were treated using the CRENBL ECP[4–8] including spin-orbit coupling and the all-electron X2C-SV(P)ALL[6–9] basis set was used for H atoms. The PBE pure functional has shown success in qualitative studies in Au nanoparticles.[10–13] The hybrid PBE0 functional was used in this work.

The anion was optimized starting at a modified structure of the $Au_{25}(SCH_3)_{18}^-$ nanocluster used in a study by Jiang *et. al.* as an initial guess.[10] In Ref. 10, Jiang *et. al.* replaced the SPET ligand in the experimental structure of $Au_{25}(SPET)_{18}^-$ with SCH_3 and then fully optimized the $Au_{25}(SCH_3)_{18}^-$ structure. We further modified the $Au_{25}(SCH_3)_{18}^-$ structure by replacing all CH_3 ligands with H atoms and the resulting $Au_{25}(SH)_{18}^-$ structure was optimized again while freezing S and Au atoms. The neutral and cationic nanocluster geometry optimizations used the optimized anion geometry as an initial guess.

Point group symmetry was determined only for the Au_{13} core with a tolerance of 0.1 Å. Double group theory notation used in the discussion of SOC orbitals were adopted from Altmann and Herzig.[14]

Table S1. Initial guess geometry for $Au_{25}(SH)_{18}^{-}$ geometry optimization. Coordinates are given in Å.

Atom	x	y	z	Atom	x	y	z
Au	-0.0000885	-0.0001091	-0.0000271	S	0.4936026	0.5482726	5.1030346
Au	0.3872277	0.8749485	2.6676833	S	-2.4731704	3.6759960	2.5867286
Au	-1.7927749	1.9966002	0.9235247	S	-4.2726270	-0.8648132	2.7767167
Au	-1.9867351	-0.8257853	1.8678465	S	2.0582931	3.4150426	-4.7588103
Au	1.1256729	2.5683654	0.4410713	S	-2.0416434	-4.6950937	-3.5362431
Au	2.6786204	0.0163060	0.9550377	S	-2.0124748	3.8112365	-2.8709241
Au	3.2316376	2.7733868	2.8382573	S	-4.5702391	-0.9141695	-2.2537504
Au	-0.7333198	-1.4585580	4.8203890	S	5.6277437	-2.6730753	0.0437644
Au	-0.1910256	4.1004554	3.0290360	S	-2.0314418	-4.7582264	0.2296970
Au	-1.9996652	-3.9994488	2.4651629	S	-0.4936003	-0.5488877	-5.1029805
Au	0.6579257	-2.0118399	1.8796801	S	2.4729317	-3.6764557	-2.5864726
Au	-3.7804909	3.1121479	-1.4556242	S	4.2727994	0.8655743	-2.7761051
Au	-4.8459218	0.8905441	1.3028838	H	-4.2280863	-0.2558318	3.9357067
Au	-0.3873230	-0.8752367	-2.6676623	H	-0.3768421	1.3635790	5.6449769
Au	1.7926868	-1.9966955	-0.9235382	H	2.1164451	4.6445526	4.8427942
Au	1.9867123	0.8256467	-1.8677623	H	0.9935753	5.5560964	-0.1879484
Au	-1.1258543	-2.5685217	-0.4409917	H	-2.9152296	4.7374549	1.9590434
Au	-2.6787687	-0.0165104	-0.9550329	H	-2.3737312	3.4130893	-4.0655256
Au	-3.2317456	-2.7733854	-2.8385324	H	1.3320099	4.3482695	-5.3224593
Au	0.7331279	1.4580758	-4.8202932	H	-4.5628704	-0.2213189	-3.3655075
Au	0.1908359	-4.1008402	-3.0290352	H	4.2287252	0.2573547	-3.9355129
Au	1.9994330	3.9991410	-2.4651176	H	0.3771322	-1.3640459	-5.6446835
Au	-0.6581093	2.0117216	-1.8796863	H	-2.1165189	-4.6444052	-4.8431189
Au	3.7805963	-3.1121927	1.4553590	H	2.9148865	-4.7378663	-1.9586322
Au	4.8459463	-0.8905056	-1.3031006	H	-0.9934490	-5.5563106	0.1880727
S	-2.0588541	-3.4152630	4.7588269	H	-1.3328350	-4.3485544	5.3227094
S	2.0414296	4.6950445	3.5359188	H	2.3738133	-3.4133239	4.0651794
S	2.0125679	-3.8114002	2.8705510	H	6.5163059	-2.1000542	0.8172009
S	4.5701741	0.9141997	2.2535404	H	4.5629669	0.2214407	3.3653556
S	-5.6277081	2.6730040	-0.0441308	H	-6.5162384	2.1000131	-0.8176264
S	2.0315195	4.7579542	-0.2296715				

Table S2. Optimized geometry of $Au_{25}(SH)_{18}^{-}$. Coordinates are given in Å.

Atom	x	y	z	Atom	x	y	z
Au	-0.000089	-0.000109	-0.000027	S	0.493603	0.548273	5.103035
Au	0.387228	0.874949	2.667683	S	-2.473170	3.675996	2.586729
Au	-1.792775	1.996600	0.923525	S	-4.272627	-0.864813	2.776717
Au	-1.986735	-0.825785	1.867847	S	2.058293	3.415043	-4.758810
Au	1.125673	2.568365	0.441071	S	-2.041643	-4.695094	-3.536243
Au	2.678620	0.016306	0.955038	S	-2.012475	3.811237	-2.870924
Au	3.231638	2.773387	2.838257	S	-4.570239	-0.914170	-2.253750
Au	-0.73332	-1.458558	4.820389	S	5.627744	-2.673075	0.043764
Au	-0.191026	4.100455	3.029036	S	-2.031442	-4.758226	0.229697
Au	-1.999665	-3.999449	2.465163	S	-0.493600	-0.548888	-5.102981
Au	0.657926	-2.011840	1.879680	S	2.472932	-3.676456	-2.586473
Au	-3.780491	3.112148	-1.455624	S	4.272799	0.865574	-2.776105
Au	-4.845922	0.890544	1.302884	H	-4.166958	-0.215086	3.959797
Au	-0.387323	-0.875237	-2.667662	H	-0.450754	1.377855	5.607032
Au	1.792687	-1.996696	-0.923538	H	2.068998	4.580966	4.884976
Au	1.986712	0.825647	-1.867762	H	0.933273	5.549256	-0.190976
Au	-1.125854	-2.568522	-0.440992	H	-2.850902	4.775192	1.892528
Au	-2.678769	-0.016510	-0.955033	H	-2.395201	3.309103	-4.069366
Au	-3.231746	-2.773385	-2.838532	H	1.239004	4.338754	-5.313825
Au	0.733128	1.458076	-4.820293	H	-4.506229	-0.182147	-3.392238
Au	0.190836	-4.100840	-3.029035	H	4.167577	0.216615	-3.959647
Au	1.999433	3.999141	-2.465118	H	0.450896	-1.378425	-5.606792
Au	-0.658109	2.011722	-1.879686	H	-2.069044	-4.580843	-4.885289
Au	3.780596	-3.112193	1.455359	H	2.850531	-4.775572	-1.892075
Au	4.845946	-0.890506	-1.303101	H	-0.933074	-5.549365	0.191099
S	-2.058854	-3.415263	4.758827	H	-1.239847	-4.339120	5.314017
S	2.041430	4.695045	3.535919	H	2.395275	-3.309451	4.069076
S	2.012568	-3.811400	2.870551	H	6.481362	-2.039272	0.881364
S	4.570174	0.914200	2.253540	H	4.506302	0.182288	3.392108
S	-5.627708	2.673004	-0.044131	H	-6.481250	2.039184	-0.881795
S	2.031520	4.757954	-0.229672				

Table S3. Optimized geometry of $Au_{25}(SH)^{0}_{18}$. Coordinates are given in Å.

Atom	x	y	z	Atom	x	y	z
Au	0.000031	0.001922	0.006890	S	-1.065412	-2.306403	-4.449236
Au	-1.180574	-1.183507	-2.242152	S	-1.573691	2.488431	-4.209168
Au	-0.522666	1.815905	-2.063322	S	3.144907	0.521280	-4.058058
Au	1.787219	-0.253989	-2.138845	S	-3.419319	3.626523	3.536226
Au	-2.714396	0.449877	-0.596096	S	4.964745	0.773423	3.478498
Au	-1.710614	-2.199461	0.434910	S	-1.227504	5.032741	0.616298
Au	-4.128165	-2.184906	-1.746561	S	3.650971	3.730183	-0.027333
Au	1.182081	-2.834752	-3.806705	S	-1.444670	-4.904543	3.327525
Au	-3.186759	0.804487	-3.666625	S	5.059514	-1.214711	0.230945
Au	4.057134	-2.372338	-1.606776	S	1.068523	2.309476	4.461684
Au	1.023439	-2.529083	-0.705221	S	1.574356	-2.499634	4.213698
Au	0.162442	4.793631	-1.319167	S	-3.146744	-0.514610	4.067801
Au	2.182547	2.642549	-3.506061	H	2.289517	0.177584	-5.087987
Au	1.180199	1.183061	2.255174	H	-0.808676	-1.189834	-5.222031
Au	0.521725	-1.811012	2.072810	H	-4.682740	-1.552098	-4.580680
Au	-1.779165	0.254913	2.152169	H	-5.095040	2.244731	-1.133993
Au	2.712989	-0.450031	0.609121	H	-2.270600	3.602250	-3.781285
Au	1.711146	2.194777	-0.423258	H	-0.237908	5.327257	1.535117
Au	4.119086	2.178367	1.735309	H	-3.131463	4.818193	2.895416
Au	-1.176330	2.834436	3.803823	H	2.979831	4.683328	0.714844
Au	3.177525	-0.807607	3.663018	H	-2.290385	-0.179421	5.099748
Au	-4.051689	2.364396	1.603614	H	0.807672	1.194222	5.234998
Au	-1.015661	2.530215	0.716285	H	4.658805	1.558527	4.574198
Au	-0.159980	-4.788310	1.315584	H	2.272002	-3.609145	3.775878
Au	-2.186161	-2.633658	3.500386	H	5.086588	-2.258324	1.136409
S	3.426837	-3.623635	-3.546846	H	3.143024	-4.820589	-2.914266
S	-4.978044	-0.770796	-3.479429	H	0.254676	-5.325421	-1.535255
S	1.240491	-5.031141	-0.612245	H	-2.596922	-5.464376	2.808434
S	-3.644943	-3.734916	0.011953	H	-2.965633	-4.681024	-0.731719
S	1.432374	4.910626	-3.340135	H	2.585069	5.479262	-2.831824
S	-5.065117	1.202800	-0.226624				

Table S4. Optimized geometry of $Au_{25}(SH)_{18}^{+}$. Coordinates are given in Å.

Atom	x	y	z	Atom	x	y	z
Au	0.000040	0.001762	-0.005347	S	1.075224	-2.214580	4.493645
Au	1.186277	-1.168036	2.249372	S	1.795289	2.537691	4.090575
Au	0.659525	1.851914	1.992543	S	-3.015472	0.787802	4.114108
Au	-1.740700	-0.108256	2.190793	S	3.483145	3.363646	-3.727755
Au	2.746888	0.343746	0.515115	S	-5.011057	0.898752	-3.376332
Au	1.598591	-2.286972	-0.407027	S	1.428197	4.952273	-0.805566
Au	4.071118	-2.318600	1.710481	S	-3.479575	3.890956	0.009216
Au	-1.208994	-2.661641	3.923031	S	1.131738	-5.062328	-3.209239
Au	3.316367	0.766652	3.560103	S	-5.113878	-0.986569	-0.064677
Au	-4.116505	-2.135639	1.781096	S	-1.078923	2.215983	-4.504037
Au	-1.115323	-2.457797	0.812212	S	-1.797642	-2.553077	-4.089143
Au	0.088186	4.833181	1.175740	S	3.014465	-0.780251	-4.123426
Au	-1.967706	2.843818	3.477591	H	-2.154053	0.434639	5.135785
Au	-1.185890	1.168087	-2.259010	H	0.887701	-1.063873	5.235631
Au	-0.657638	-1.847257	-1.999092	H	4.727984	-1.627713	4.508963
Au	1.731906	0.109157	-2.201103	H	5.222488	2.039347	0.935990
Au	-2.745385	-0.344590	-0.525342	H	2.530341	3.605628	3.612507
Au	-1.598998	2.282091	0.399243	H	0.424824	5.260274	-1.704797
Au	-4.060408	2.312336	-1.695273	H	3.261887	4.583676	-3.114375
Au	1.202501	2.661889	-3.917706	H	-2.787908	4.792614	-0.777138
Au	-3.305444	-0.770884	-3.553724	H	2.149018	-0.436969	-5.145084
Au	4.109767	2.129134	-1.775557	H	-0.886677	1.065555	-5.245239
Au	1.107533	2.459264	-0.819624	H	-4.694017	1.636417	-4.501450
Au	-0.088989	-4.827669	-1.167984	H	-2.533872	-3.614094	-3.597608
Au	1.972499	-2.834585	-3.468200	H	-5.213958	-2.053632	-0.937070
S	-3.491798	-3.357779	3.741472	H	-3.276069	-4.583555	3.137834
S	5.029258	-0.894022	3.377007	H	-0.446351	-5.259124	1.708532
S	-1.443944	-4.949362	0.803424	H	2.270769	-5.661176	-2.704707
S	3.472630	-3.896313	0.013102	H	2.771721	-4.788246	0.802280
S	-1.112684	5.066772	3.228540	H	-2.250586	5.679640	2.738679
S	5.118682	0.974372	0.061424				

S3 Molecular Orbitals

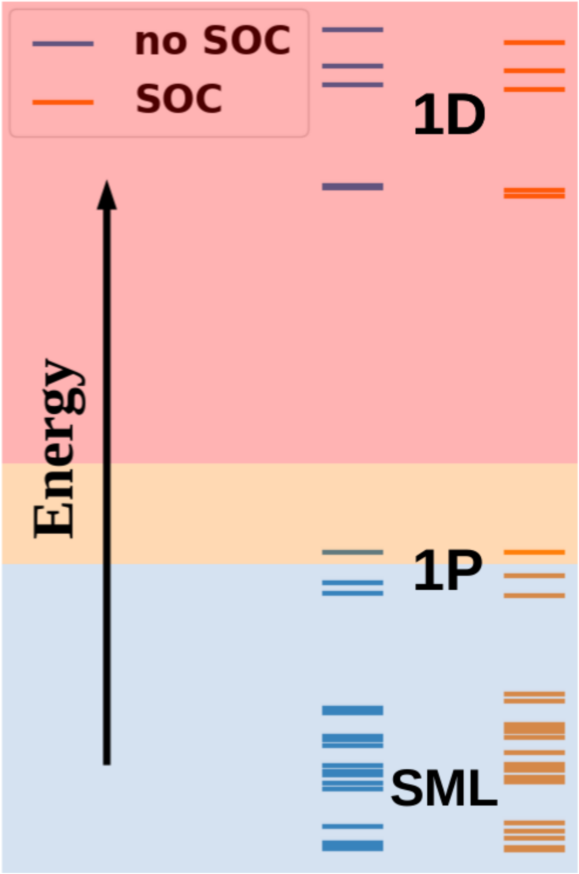

Figure S1. Molecular orbital diagram depicting the effects of SOC in neutral Au_{25}. The blue-shaded region are doubly-occupied orbitals, orange-shaded region are singly-occupied orbitals, and the red-shaded region are unoccupied orbitals. Orbital energies were shifted such that the highest energy $1P$ were aligned.

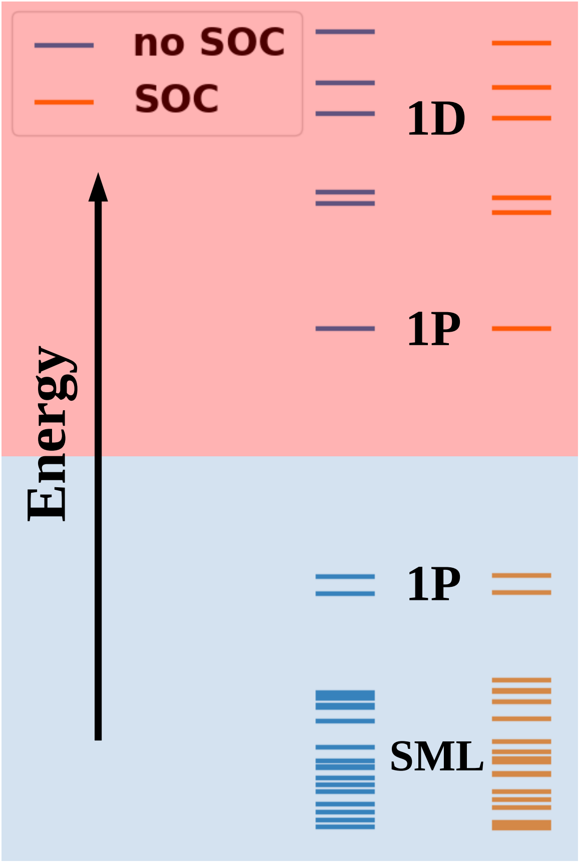

Figure S2. Molecular orbital diagram depicting the effects of SOC in the Au_{25} cation. The blue-shaded region are doubly-occupied orbitals and the red-shaded region are unoccupied orbitals. Orbital energies were shifted such that the highest energy $1P$ were aligned.

Table S5. Atomic orbital character of $Au_{25}(SC_6H_{13})_{18}^-$ superorbitals from calculation with SOC. Orbitals are arranged down the table with increasing energy.

Superorbital	s	p	d
$1P_1$	25.64%	39.86%	34.50%
$1P_2$	24.17%	39.28%	36.55%
$1P_3$	22.92%	38.55%	38.55%
$1D_1$	41.50%	37.92%	20.56%
$1D_2$	40.87%	37.87%	21.25%
$1D_3$	40.22%	34.48%	25.30%
$1D_4$	39.44%	33.14%	27.42%
$1D_5$	40.36%	32.26%	27.38%

S4 $Au_{25}(SPET)^-_{18}$ UV-Vis Fingerprint Spectrum

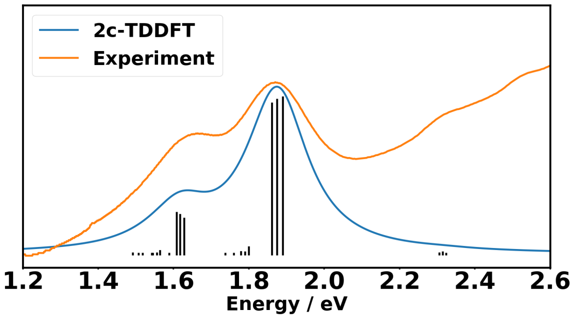

Figure S3. Experimental and computed spectra of $Au_{25}(SR)^-_{18}$. The excited states obtained from 2C-TDDFT are plotted as black lines. Spectra were generated by applying Lorentzian broadening to the excited states with a half-width at half max of 0.095 eV. The computed spectrum is compared to the experimental spectrum of $Au_{25}(SPET)^-_{18}$. Spectrum was recorded at 78 K.

References

(1) Zhu, M.; Lanni, E.; Garg, N.; Bier, M. E.; Jin, R. Kinetically Controlled, High-Yield Synthesis of Au_{25} Clusters. *J. Am. Chem. Soc.* **2008**, *130*, 1138 – 1139.

(2) Zhu, M.; Aikens, C. M.; Hendrich, M. P.; Gupta, R.; Qian, H.; Schatz, G. C.; Jin, R. Reversible Switching of Magnetism in Thiolate-Protected Au_{25} Superatoms. *J. Am. Chem. Soc.* **2009**, *131*, 2490 – 2492.

(3) Liu, Z.; Zhu, M.; Meng, X.; Xu, G.; Jin, R. Electron Transfer between $[Au_{25}(SC_2H_4Ph)_{18}]^{-}TOA^{+}$ and Oxoammonium Cations. *J. Am. Chem. Soc.* **2009**, *131*, 2490 – 2492.

(4) Pacios, L. F.; Christiansen, P. A. Ab Initio Relativistic Effective Potentials with Spin-orbit Operators. I. Li through Ar. *J. Chem. Phys.* **1984**, *82*, 2664–2671.

(5) Ross, R. B.; Powers, J. M.; Atashroo, T.; C., E. W.; LaJohn, L. A.; Christiansen, P. A. Ab Initio Relativistic Effective Potentials with Spin-orbit Operators. IV. Cs through Rn. *J. Chem. Phys.* **1990**, *93*, 6654–6670.

(6) Schuchardt, K. L.; Didier, B. T.; Elsethagen, T.; Sun, L.; Gurumoorthi, V.; Chase, J.; Li, J.; Windus, T. L. Basis Set Exchange: A Community Database for Computational Sciences. *J. Chem. Inf. Model.* **2007**, *47*, 1045–1052.

(7) Feller, D. The Role of Databases in Support of Computational Chemistry Calculations. *J. Comput. Chem.* **1996**, *17*, 1571–1586.

(8) Pritchard, B. P.; Altarawy, D.; Didier, B.; Gibson, T. D.; Windus, T. L. New Basis Set Exchange: An Open, Up-to-Date Resource for the Molecular Sciences Community. *J. Chem. Inf. Model.* **2019**, *59*, 4814–4820.

(9) Pollak, P.; Weigand, F. Segmented Contracted Error-Consistent Basis Sets of Double-

and Triple-ζ Valence Quality for One- and Two-Component Relativistic All-Electron Calculations. *J. Chem. Theory Comput.* **2017**, *13*, 3696–3705.

(10) Jiang, D.-e.; Kühn, M.; Tang, Q.; Weigand, F. Superatomic Orbitals under Spin–Orbit Coupling. *J. Phys. Chem. Lett.* **2014**, *5*, 3286–3289.

(11) Lopez-Acevedo, O.; Tsunoyama, H.; Tsukuda, T.; Hannu, H.; Aikens, C. M. Chirality and Electronic Structure of the Thiolate-Protected Au_{38} Nanocluster. *J. Am. Chem. Soc.* **2010**, *132*, 8210–8218.

(12) Tlahuice-Flores, A.; Whetten, R. L.; Jose-Yacaman, M. Ligand Effects on the Structure and the Electronic Optical Properties of Anionic $Au_{25}(SR)_{18}$ Clusters. *J. Phys. Chem. C* **2013**, *117*, 20867–20875.

(13) Akola, J.; Walter, M.; Whetten, R. L.; Häkkinen, H.; Grönbeck, H. On the Structure of Thiolate-Protected Au_25. *J. Am. Chem. Soc.* **2008**, *130*, 3756–3757.

(14) Altmann, S. L.; Herzig, P. *Point-Group Theory Tables*; Clarendon Press, 2011.
